# Resistance Training and Lymphedema in Breast Cancer Survivors

**DOI:** 10.1001/jamanetworkopen.2025.14765

**Published:** 2025-06-11

**Authors:** Parisa Shamsesfandabadi, Mostafa Shams Esfand Abadi, Yue Yin, David J. Carpenter, Chris Peluso, Christie Hilton, Suzanne B. Coopey, Janette Gomez, Sushil Beriwal, Colin E. Champ

**Affiliations:** 1 Department of Radiation Oncology, Allegheny Health Network, Pittsburgh, Pennsylvania; 2 Department of Statistical Sciences, Wake Forest University, Winston-Salem, North Carolina; 3 Allegheny-Singer Research Institute, Allegheny Health Network, Pittsburgh, Pennsylvania; 4 Department of Radiation Oncology, Wellstar Paulding Medical Center, Hiram, Georgia; 5 Allegheny Health Network Cancer Institute Exercise Oncology and Resiliency Center, Pittsburgh, Pennsylvania; 6 Department of Medical Oncology, Allegheny Health Network, Pittsburgh, Pennsylvania; 7 Department of Surgery, Allegheny Health Network, Pittsburgh, Pennsylvania

## Abstract

**Importance:**

Lymphedema is a common treatment-related adverse effect among breast cancer survivors that can limit activity and mobility. Although exercise is associated with improved outcomes after breast cancer treatment, data are limited on the association of intense physical activity, including strength training, with lymphedema.

**Objective:**

To examine the association between resistance training and lymphedema among breast cancer survivors.

**Design, Setting, and Participants:**

In this cohort study conducted between September 15, 2022, and March 26, 2024, women from 3 prospective studies (EXERT-BC, EXERT-BCN, and EXERT-C) underwent thrice-weekly, dose-escalated resistance training for 3 months to promote strength, mobility, and hypertrophy.

**Main Outcomes and Measures:**

Lymphedema was assessed at baseline and completion using bioimpedance analysis to evaluate fluid and body composition metrics, distinguishing among intracellular water, extracellular water, and total body water.

**Results:**

A total of 115 women completed the exercise regimen (median age, 54 years; range, 24-71 years). Ninety-six participants (83%) underwent sentinel lymph node biopsy, whereas 14 (12%) underwent axillary lymph node dissection. At baseline, 15 women (13%) had clinical lymphedema: 8 (8%) in the sentinel lymph node biopsy group and 7 (37%) in the axillary lymph node dissection group. No participants experienced subjective or clinical worsening of lymphedema after completing the exercise regimen. Bilateral arm lean mass significantly increased after resistance training (affected arm: median [IQR], 5.64 [4.98-6.20] lb; 95% CI, 5.40-5.84 lb) vs baseline (median [IQR], 5.45 [4.92-6.08] lb; 95% CI, 5.34-5.67 lb) (*s* = 1789.5; *P* < .001). The edema index (extracellular water to total body water ratio) significantly improved in all patients, signifying a reduction in lymphedema at the completion of exercise (mean, 0.383; 95% CI, 0.382-0.385) compared with baseline (mean, 0.385; 95% CI, 0.384-0.386) (*t*_110_ = 4.05; *P* < .001).

**CONCLUSIONS AND RELEVANCE:**

In this cohort study of breast cancer survivors, intense resistance training did not exacerbate lymphedema and was associated with improvements in fluid balance and lean mass in the upper extremities. These findings suggest support for the inclusion of structured resistance exercise as part of breast cancer treatment and survivorship care.

## Key Points

**Question:**

Is intense resistance training associated with lymphedema in breast cancer survivors?

**Findings:**

In this cohort study of 115 breast cancer survivors, intense resistance training was not associated with an increase in lymphedema symptoms. Bioimpedance analysis revealed significant reductions in extracellular water and improved fluid balance, suggesting a potential therapeutic benefit.

**Meaning:**

These findings suggest support for the safety and potential benefits of intense resistance training for breast cancer survivors, challenging outdated restrictions on strength training.

## Teaser

This cohort study of breast cancer survivors assesses whether resistance training is associated with lymphedema.

## Introduction

Lymphedema remains a common sequela of breast cancer treatment, with rates ranging from 3% to 8% after sentinel lymph node biopsy (SLNB) to more than 25% after axillary lymph node dissection (ALND).^1^ Although radiation therapy may further increase the risk of lymphedema, this risk is strongly associated with the extent of surgical dissection. Obesity is strongly associated with lymphedema and is the only modifiable risk factor.^2^

Optimal body composition, including low adipose tissue and high muscle mass, is associated with improved survival rates among women treated for breast cancer, as are increased activity levels before and after breast cancer diagnosis and treatment.^3,4^ Women treated for breast cancer also experience significantly increased risks of dying of metabolic disease compared with the general population.^5^

Resistance training may provide multiple benefits in the breast cancer population by increasing muscle mass, decreasing adipose tissue, and improving metabolism, bone density, strength, and mobility.^6^ Thus, considerable efforts are under way to include exercise, and specifically resistance training, as part of the treatment of breast cancer and during survivorship. However, to optimize these outcomes, an effective dose of resistance training must be met, and prior work often did not meet this threshold.^7^

Despite the many benefits of resistance training, there remains a dearth of research on the effects of intense and dose-escalated physical activity, particularly strength training achieving optimal thresholds, on lymphedema development or exacerbation in this population. The fear of inducing or worsening lymphedema has historically led to cautious recommendations regarding exercise, which may have resulted in underwhelming results from exercise studies in breast cancer populations.^7^

Although lymphedema has traditionally been monitored with serial circumferential arm measurements, the accuracy of this technique remains in question, particularly in individuals who are exercising and modifying their muscle and fat mass in the upper extremities.^8^ Bioimpedance analysis (BIA) is a newer method to accurately measure lymphedema based on fluid levels as opposed to circumferential arm measurements, while providing data on upper extremity adipose tissue and muscle mass.^9^ However, there are limited data assessing changes in bioimpedance limb measurements in a large group of breast cancer survivors engaging in an intense exercise regimen monitored under close supervision to ensure adherence, accuracy, and efficacy. We therefore assessed whether intense resistance training was associated with lymphedema in breast cancer survivors by measuring intracellular and extracellular fluid levels in the upper extremities of patients with breast cancer before and after intense exercise interventions.

## Methods

A previous study reported the safety and efficacy of dose-escalated resistance training in a breast cancer population with no increase in clinical lymphedema.^10^ In the current study, we conducted a more comprehensive analysis of the association with lymphedema by measuring intracellular and extracellular fluid levels in the upper extremities of patients with breast cancer before and after intense exercise interventions using resistance training at our exercise oncology center delivered in 3 prospective study cohorts: EXERT-BC,^11^ EXERT-BCN,^12^ and EXERT-C.^13^ Written informed consent was obtained for each participant. This study was approved by the Allegheny Health Network Review Board. This prospective cohort study follows the Strengthening the Reporting of Observational Studies in Epidemiology (STROBE) reporting guidelines for cohort studies.^14^

Women aged 20 to 89 years with biopsy-proven ductal carcinoma in situ or invasive breast cancer were eligible for one of these trials. Data on race were collected to help assess the applicability of research findings. These data were obtained through both self-reporting in the digital medical records and recording by the investigators. Participants were required to be able to get up and down from the ground, squat their body weight, and participate in group exercise. Individuals with severe arthritic, joint, cardiovascular, or musculoskeletal condition deemed unsafe to engage in resistance training were excluded. Participants actively undergoing systemic cytotoxic chemotherapy were excluded from EXERT-BC and EXERT-BCN but were permitted on EXERT-C. Radiation therapy, antiestrogen therapy, and targeted systemic therapy were permitted on all studies. Participants were screened by study personnel at the time of oncologic consultation or follow-up. Treatment and medical records were manually curated. Recruitment occurred between September 15, 2022, and March 26, 2024, at the Allegheny Health Network departments of surgical, medical, and radiation oncology, along with the Allegheny Health Network Cancer Institute Exercise Oncology and Resiliency Center.

All participants were enrolled in a 3-month, thrice-weekly, dose-escalated exercise regimen using multijoint compound movements following linear progression balanced with resistance training exercises with an adequate volume to promote hypertrophy. These workouts have been previously described and followed a similar exercise course for all participants.^10,15^ An outline of the program structure is listed in Table 1. The objectives of the studies were to assess safety, adherence, and the association with body composition, strength, mobility, and quality of life during and after breast cancer treatment. For body composition measurements, each participant underwent body composition analysis via a BIA machine (InBody 970, InBody Co) 1 week prior to start of the exercise regimen and within 1 week of completion. Ultrasonography was used as a second measurement to confirm changes over differing modalities, with fat mass, fat-free mass, and percentage of body fat calculated using the Jackson & Pollock calculation (BodyMetrix) and measurements at the triceps, suprailiac, abdominal, and thigh area.^16^ Adherence was monitored through electronic attendance logs and direct trainer supervision. Participants were required to attend at least 75% of scheduled sessions to be considered adherent.

**Table 1.** Outline of the Exercise Program

Exercise	No. of sets/repetitions
**Monday**	
Split squat	4/8
Side planks	4 (20 s each)
Bird dog row	3/10
1-Leg glute bridge	3/10
Half-kneeling shoulder press	3/10
Biceps dumbbell curls	3/10
**Wednesday**	
Goblet squats	4/8
Band pull aparts	3/10
Hip thrust on 12-in plyo box	3/10
Incline dumbbell press	3/10
Dumbbell skull crushers	3/10
Dumbbell external rotation on knee	3/10
**Friday**	
Hex bar deadlift	4/8
TRX row	3/10
Box step-ups	3/10
Pushups	3/10
Suitcase carry	3/10
2-Leg calf raises	3/10

### Lymphedema

All participants underwent evaluation by a physical or occupational therapist specializing in lymphedema therapy at our oncology rehabilitation program. The threshold for a lymphedema diagnosis was set low to capture those with subclinical lymphedema. Lymphedema was defined as an increase in arm circumference discrepancy greater than 3% compared with the preoperative ipsilateral arm measurements. All clinically symptomatic patients received standard lymphedema care, including manual lymphatic drainage therapy and compression sleeve use, as recommended by their treating therapist. The study was designed to assess both lymphedema progression in affected individuals and risk mitigation in the broader breast cancer population. Additionally, BIA was used to assess lymphedema by providing measurements of fluid and muscle-fat metrics to distinguish between the intracellular water (ICW) and extracellular water (ECW) compartments, along with total body water (TBW).^8,17^ The edema index, based on the ECW/TBW ratio, enabled the detection of fluid imbalances associated with lymphedema development or progression. Lastly, the ratio of the affected upper extremity to the contralateral limb was calculated. These values were assessed and calculated before exercise initiation and at completion.

### Statistical Analysis

Descriptive statistics were generated to characterize the variables of interest. In cases in which the assumption of normal distribution was violated, the Wilcoxon signed-rank test was applied to compare the median rank between the first and last tests across different variables. Conversely, for variables following a normal distribution, the paired *t* test was used for the mean comparison. All statistical analyses were conducted using SAS statistical software version 9.4 (SAS Institute) using an α = .05. Benjamini-Hochberg correction was conducted to adjust *P* values. Two-sided *P* values are reported.

## Results

In total, 115 women completed 1 of 3 studies; 5 (4%) were Black and 110 (96%) were White. Adherence was high, with 10 participants (9%) not meeting threshold and a mean (SD) of 3.17 (3.15) missed days per participant. Median (range) age was 54 (24-71) years, and 15 women (13%) had clinical lymphedema at study enrollment based on arm measurements (Table 2); 8 (8%) in the SLNB group and 7 (37%) in the ALND group. Of note, 5 patients within the ALND group underwent initial SLNB and returned to the operating room for full axillary dissection. Four patients underwent bilateral surgery, limiting contralateral arm comparison assessments; thus, fluid assessment was reported for 111 participants (Table 3). Use of compression sleeves was recorded, with 13 of 15 patients (85%) with clinical lymphedema using them regularly, whereas the remaining 2 (13%) declined sleeve use due to discomfort. None of the participants underwent surgical treatment for existing lymphedema during the study period. One participant underwent a lymphovenous anastomosis procedure, and another underwent lymph node transfer, both prior to initiating the exercise regimen.

**Table 2.** Characteristics of the Study Participants

Characteristic	No. (%) of participants^a^ (N = 115)
Age, median (range), y	54 (24-71)
Disease site	
Left	57 (50)
Right	54 (47)
Bilateral	4 (3)
Stage	
DCIS	4 (3)
I	65 (57)
II	28 (24)
III	12 (10)
IV	2 (2)
Recurrence	4 (3)
Subtype	
Hormonally positive, *ERBB2*−	86 (75)
*ERBB2*^+^	15 (13)
Triple negative	14 (12)
Race	
Black	5 (4)
White	110 (96)
Menopausal status	
Premenopausal	32 (28)
Postmenopausal	83 (72)
Surgery	
Lumpectomy	69 (60)
Mastectomy	46 (40)
Lymph node surgery	
SLNB	96 (83)
ALND	14 (12)
Both	5 (4)
Patients with clinical lymphedema	15 (13)
Systemic therapy	
Endocrine therapy	75 (65)
Chemotherapy	6 (5)
Targeted therapy	15 (13)
Immunotherapy	6 (5)

Abbreviations: ALND, axillary lymph node dissection; DCIS, ductal carcinoma in situ; SLNB, sentinel lymph node biopsy.

^a^ Unless otherwise indicated.

**Table 3.** Fluid Assessment for 111 Study Patients

Variable	Body composition at baseline, median (IQR)	Body composition after exercise, median (IQR)	*P* value
Lean mass, lb			
Affected arm	5.44 (4.90-5.99)	5.59 (4.95-6.11)	<.001
Unaffected arm	5.51 (4.83-6.03)	5.53 (5.01-6.12)	<.001
TBW, lb			
Affected arm	4.25 (3.80-4.66)	4.36 (3.87-4.75)	<.001
Unaffected arm	4.29 (3.77-4.71)	4.30 (3.89-4.76)	<.001
ICW, lb			
Affected arm	2.62 (2.35-2.89)	2.69 (2.39-2.95)	<.001
Unaffected arm	2.67 (2.34-2.89)	2.67 (2.44-2.95)	<.001
ECW, lb			
Affected arm	1.62 (1.45-1.76)	1.67 (1.46-1.82)	<.001
Unaffected arm	1.63 (1.43-1.81)	1.63 (1.46-1.82)	.005
ECW/TBW ratio			
Total	0.385 (0.379-0.390)	0.383 (0.378-0.388)	.002
Αffected arm	0.381 (0.377-0.385)	0.380 (0.376-0.384)	<.001
Unaffected arm	0.381 (0.378-0.383)	0.379 (0.376-0.382)	<.001

Abbreviations: ECW, extracellular water; ICW, intracellular water; TBW, total body water.

SI conversion factor: To convert lb to kg, multiply by 0.45.

No patient experienced a self-described or clinical worsening of lymphedema at the conclusion of the study. Bilateral upper extremity lean mass of the affected arm increased significantly at the completion of exercise (median [IQR], 5.64 [4.98-6.20] lb; 95% CI, 5.40-5.84 lb) (to convert lb to kg, multiply by 0.45) compared with baseline (median [IQR], 5.45 [4.92-6.08] lb; 95% CI, 5.34-5.67 lb) (*s* = 1789.5; *P* < .001). Bilateral upper extremity lean mass of the unaffected arm increased significantly at completion of exercise (median [IQR], 5.53 [5.05-6.20] lb; 95% CI, 5.40-5.89 lb) compared with baseline (median [IQR], 5.51 [4.83-6.06] lb; 95% CI, 5.31-5.73 lb) (*s* = 1315; *P* < .001). Bilateral upper extremity TBW of the affected and unaffected arms increased significantly at the completion of exercise (affected arm: median [IQR], 4.39 [3.88-4.81] lb; 95% CI, 4.21-4.54 lb; unaffected arm: median [IQR], 4.30 [3.92-4.83] lb; 95% CI, 4.19-4.59 lb) compared with baseline (affected arm: median [IQR], 4.26 [3.81-4.76] lb; 95% CI, 4.17-4.41 lb) (*s* = 1715.5; *P* < .001; unaffected arm: median [IQR], 4.30 [3.77-4.72] lb; 95% CI, 4.14-4.45 lb) (*s* = 1241; *P* < .001). Bilateral upper extremity ICW of the affected and unaffected arms also increased significantly at the completion of exercise (affected arm: median [IQR], 2.71 [2.40-3.00] lb; 95% CI, 2.62-2.82 lb; unaffected arm: median [IQR], 2.67 [2.45-2.98] lb; 95% CI, 2.60-2.84 lb) compared with baseline (affected arm: median [IQR], 2.62 [2.36-2.91] lb; 95% CI, 2.58-2.73 lb) (*s* = 1665; *P* < .001; unaffected arm: median [IQR], 2.67 [2.34-2.93] lb; 95% CI, 2.56-2.76 lb) (*s* = 1351.5; *P* < .001). Moreover, bilateral upper extremity median ECW of the affected and unaffected arms increased significantly at the completion of exercise (affected arm: median [IQR], 1.68 [1.48-1.85] lb; 95% CI, 1.59-1.74 lb; unaffected arm: median [IQR], 1.65 [1.48-1.85] lb; 95% CI, 1.59-1.72 lb) compared with baseline (affected arm: median [IQR], 1.63 [1.46-1.81] lb; 95% CI, 1.59-1.68 lb) (*s* = 1266.5; *P* < .001; unaffected arm: median [IQR], 1.63 [1.43-1.83] lb; 95% CI, 1.57-1.70 lb) (*s* = 810.5; *P* = .004).

The bilateral edema index of the upper extremities significantly improved in all participants, signifying a reduction in lymphedema at the completion of exercise (mean, 0.383; 95% CI, 0.382-0.385) compared with baseline (mean, 0.385; 95% CI, 0.384-0.386) (*t*_110_ = 4.05, *P* < .001). There was no significant change in the ratio of ECW of the affected to unaffected arm.

Subset analysis of women who underwent SLNB is detailed in Table 4. The bilateral edema index (Figure) at the completion of exercise (mean, 0.383; 95% CI, 0.382-0.385) compared with baseline (mean, 0.385; 95% CI, 0.383-0.386) (*t*_91_ = −3.46; *P* = .002) significantly improved in women who underwent SLNB, signifying a reduction in lymphedema. In women who underwent ALND (n = 19), lean mass, ICW, ECW, and TBW of the affected and unaffected arm increased after exercise but did not show statistically significant change in this subgroup. Although body weight decreased by a median (IQR) of 0.32 (−2.35 to 0.50) kg , body fat decreased by a median (IQR) of 1.13 (0.14-2.85) kg and muscle mass increased by a median (IQR) of 0.32 (−0.09 to 0.91) kg. There were no protocol-limiting injuries during the study.

**Table 4.** Fluid Assessment for the 92 Patients in the Sentinel Lymph Node Biopsy Group

Variable	Body composition at baseline, median (IQR)	Body composition after exercise, median (IQR)	*P* value
Lean mass, lb			
Affected arm	5.44 (4.90-5.99)	5.59 (4.95-6.11)	<.001
Unaffected arm	5.51 (4.83-6.03)	5.53 (5.01-6.12)	<.001
TBW, lb			
Affected arm	4.25 (3.80-4.66)	4.36 (3.87-4.75)	<.001
Unaffected arm	4.29 (3.77-4.71)	4.30 (3.89-4.76)	<.001
ICW, lb			
Affected arm	2.62 (2.35-2.89)	2.69 (2.39-2.95)	<.001
Unaffected arm	2.67 (2.34-2.89)	2.67 (2.44-2.95)	<.001
ECW, lb			
Affected arm	1.62 (1.45-1.76)	1.67 (1.46-1.82)	<.001
Unaffected arm	1.63 (1.43-1.81)	1.63 (1.46-1.82)	.005
ECW/TBW ratio			
Total	0.385 (0.379-0.390)	0.383 (0.378-0.388)	.002
Affected arm	0.381 (0.377-0.385)	0.380 (0.376-0.384)	<.001
Unaffected arm	0.381 (0.378-0.383)	0.379 (0.376-0.382)	<.001

Abbreviations: ECW, extracellular water; ICW, intracellular water; TBW, total body water.

SI conversion factor: To convert lb to kg, multiply by 0.45.

**Figure.**
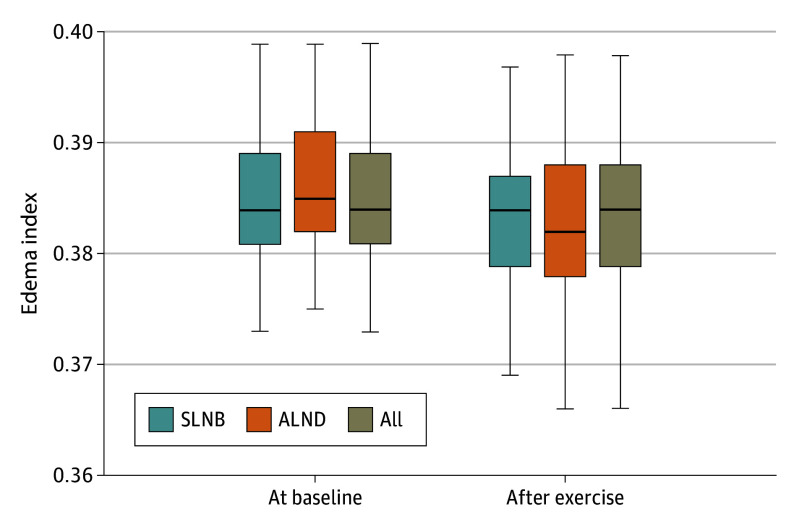
Mean Bilateral Edema Indexes at Exercise Completion Compared With Baseline Error bars represent 95% CIs. ALND indicates axillary lymph node dissection; SLNB, sentinel lymph node biopsy.

## Discussion

Women who underwent an intense exercise regimen focused on strength training and hypertrophy experienced significant increases in bilateral upper extremity muscle mass. Additionally, no increase in the ratio of extracellular fluid to total water content of the upper extremity was experienced; conversely, this ratio was reduced. The latter, which is the amount of third-spaced fluid in the upper extremity, represents a reduction in lymphedema. These findings highlight the safety of strength and resistance training in a large group of patients with breast cancer during and after treatment and illustrate its potential as a reduction strategy for lymphedema.

To our knowledge, this is one of the largest studies assessing a strictly prescribed, dose-escalated, and observed resistance training regimen in a large population of women with breast cancer. Methods to increase muscle mass in women during and after breast cancer treatment remain important because muscle wasting and weakness are common sequelae of cancer treatment and physical inactivity and low muscle mass is associated with worse outcomes.^3^ By promoting muscle hypertrophy, strength, and functional capacity, strength training may mitigate the functional impairments and muscle loss associated with breast cancer treatment, thereby improving overall quality of life and physical function. However, reaching an appropriate dose of exercise to produce optimal neurologic and muscular adaption to promote considerable hypertrophy requires a program to exceed an appropriate threshold and quantity that are often not met.^7,18^

In our cohort, the affected arm of women experienced a lower ratio of extracellular to total fluid levels after 3 months of intense resistance training, signifying a decrease in lymphedema. The favorable changes in fluid dynamics, evidenced by reductions in extracellular water and improvements in fluid balance, suggest potential benefits for lymphedema management and symptom alleviation. These changes also were seen in women who underwent axillary dissection, although the numbers were small. In addition, 40% of participants underwent mastectomy, illustrating that many participants were at higher risk regarding lymphedema and subsequent mobility and strength deficits from their treatment. Physiologically, increased muscle mass and contraction of this muscle mass during resistance training and exercise would be expected to aid in fluid return through the lymphatic system of the upper extremities. Other data have revealed the safety of resistance training on lymphedema, showing no increase or even potentially improved lymphedema analyzed via circumference measurements, BIA, or clinically.^19,20^ However, it should be noted than an effective resistance training program that increases upper extremity muscle mass may invalidate traditional serial circumferential arm measurements due to asymmetric hypertrophy that can occur during exercise training.^21^ A prior assessment of aerobic and resistance exercise revealed no improvement in self-esteem, physical fitness, body composition, and chemotherapy completion rate but did reveal a significantly improved cancer-specific quality of life in patients with breast cancer receiving chemotherapy without causing lymphedema or significant adverse events.^22^ In breast cancer survivors with lymphedema, slowly progressive weightlifting had no significant effect on limb swelling and resulted in a decreased incidence of exacerbations of lymphedema, reduced symptoms, and increased strength.^23^

### Limitations

There are limitations to our study. One major limitation is the lack of a control group. This study was a single-arm cohort study; thus, we could not directly compare the effects of exercise to the natural progression of lymphedema in a nonexercise group. Randomized clinical trials are needed to validate these findings and provide a clearer understanding of the impact of resistance training on lymphedema outcomes.

Another limitation is the short duration of follow-up. The intervention lasted 3 months, and although it demonstrated significant improvements in lean mass and fluid balance, the long-term sustainability of these benefits remains uncertain. Future studies should assess whether ongoing resistance training is necessary to maintain these improvements and whether lymphedema outcomes change during extended periods.

Additionally, patient-reported outcomes were not included in this study. Although objective measures, such as BIA and circumferential arm measurements, were used, self-reported symptom burden and functional limitations were not collected. Including patient-reported outcomes in future research would provide a more comprehensive understanding of the patient experience and the functional impact of resistance training.

Potential confounding variables may have also influenced the results. Factors such as diet, medication use, and baseline physical activity levels were not controlled for and could have contributed to the observed outcomes. Although adherence to the exercise regimen was high, variations in individual metabolic responses or lifestyle factors may have affected fluid balance and body composition changes.

Additionally, while the overall cohort size (n = 115) was robust, the ALND subgroup (n = 19) was relatively small, which may have limited the statistical power for certain subgroup analyses. Future studies with larger ALND cohorts are needed to better assess the effects of resistance training in this high-risk population. These limitations highlight areas for future research, particularly the need for longer-term studies, randomized clinical trials, and inclusion of patient-reported outcomes to fully understand the role of resistance training in lymphedema management.

## Conclusions

In a large cohort of women engaging in an intense and prescribed strength training program that promotes upper extremity hypertrophy, lymphedema in breast cancer survivors was not exacerbated and improved based on fluid measurements. These findings support the safety of integrating effective resistance training as part of the treatment of breast cancer and during survivorship.

## Notes

Supplement.
Data Sharing Statement
