# Supplementary material for: Resistance Training and Lymphedema in Breast Cancer Survivors
Source: JAMA Netw Open. 2025 Jun 11;8(6):e2514765. doi: 10.1001/jamanetworkopen.2025.14765 (PMC12159776; doi:10.1001/jamanetworkopen.2025.14765)
Supplement: Supplement. — Data Sharing Statement [file jamanetwopen-e2514765-s001.pdf]

Data Sharing Statement

Shamsesfandabadi. Resistance Training and Lymphedema in Breast Cancer Survivors. *JAMA Netw Open*. Published June 11, 2025. doi:10.1001/jamanetworkopen.2025.14765

Data
Data available: No

Additional Information
Explanation for why data not available: data may be available upon contacting the authors
